# Infants discriminate the source of social touch at stroking speeds eliciting maximal firing rates in CT-fibers

**DOI:** 10.1016/j.dcn.2019.100639

**Published:** 2019-03-19

**Authors:** Marie Aguirre, Auriane Couderc, Justine Epinat-Duclos, Olivier Mascaro

**Affiliations:** Institute for Cognitive Sciences Marc Jeannerod, CNRS UMR 5304/Univ Lyon, Bron, France

**Keywords:** Infancy research, Touch, Social cognition, Social grooming, CT fibers, Caregiving

## Abstract

The evaluation of interpersonal touch is heavily influenced by its source. For example, a gentle stroke from a loved one is generally more pleasant than the same tactile stimulation from a complete stranger. Our study tested the early ontogenetic roots of humans’ sensitivity to the source of interpersonal touch. We measured the heart rate of three groups of nine-month-olds while their legs were stroked with a brush. The participants were stroked at a different speed in each group (0.3 cm/s, 3 cm/s, 30 cm/s). Depending on the Identity condition (stranger vs. parent), the person who acted as if she was stroking the infant’s leg was either an unfamiliar experimenter or the participant’s caregiver. In fact, the stimulation was always delivered by a second experimenter blind to the Identity condition. Infants’ heart rate decreased more in reaction to strokes when their caregiver rather than a stranger acted as the source of the touch. This effect was found only for tactile stimulations whose velocity (3 cm/s) is known to elicit maximal mean firing rates in a class of afferents named C-tactile fibers (CTs). Thus, the infants’ reaction to touch is modulated not just by its mechanical properties but also by its social source.

## Highlights

• Infants’ cardiac response to touch varies depending on its social source.
• This effect occurs only for velocities yielding maximal firing rates in CTs.
• Infants’ responses to touch do not just depend upon its mechanical properties.

## 1 Introduction

Tactile contact plays an obvious role in many social interactions such as greetings, sex, comfort and physical aggression. A light interpersonal touch can also elicit positive feelings, generosity, and compliance (Crusco and Wetzel, 1984; Fisher et al., 1976; Guéguen, 2002; Guéguen and Fischer-Lokou, 2002, 2003; Hornik, 1992; Willis and Hamm, 1980). Because of the role of tactile stimulation in regulating social interactions and social relationships, interpersonal touch is often processed in very different ways depending on its source. In human adults, the total bodily area where touch is considered acceptable or pleasant is larger for closer relationships (Heslin et al., 1983; Jourard, 1966; Suvilehto et al., 2015). Similarly, heterosexual men rate the same tactile stimulation as more pleasant when they think that the person touching them is an attractive woman rather than another man (Gazzola et al., 2012; Scheele et al., 2014). In short, human adults do not treat interpersonal touch just as a mechanical event, but also as a social one, whose socio-cognitive evaluation depends on the source of the touch. Here, we probe the underpinnings of the sensitivity to the source of interpersonal touch during infancy, focusing on positive affective touch processing, via the preferential activation of C Tactile fibers (henceforth, CTs).

Interpersonal touch regulates social relationships from infancy onward. Tactile stimulation is a part of the typical repertoire of mammalian caregiving behaviors (Feldman, 2011), and touching and being touched is crucial for bodily and neuro-cognitive development in humans and in non-human primates (Brauer et al., 2016; Bremner and Spence, 2017; Cascio et al., 2019; Feldman et al., 2014; Field, 2010, 2014; Harlow and Zimmermann, 1958; Reece et al., 2016; Seidl et al., 2015; Simpson et al., 2017; Suomi, 1995). Interpersonal touch reduces infants’ response to stress (Feldman et al., 2010; Stack and Muir, 1990, 1992) and enhances social learning (Della Longa et al., 2017). Furthermore, the quality of tactile interactions between caregivers and infants has important long-term social consequences, influencing the formation of bonds and attachment behaviors throughout the lifespan (Feldman, 2011; Field, 2014; Hertenstein, 2002; Hofer, 1987, 1995). Thus, interpersonal touch is a crucial medium for mammalian infants to react adaptively to their environment. Therefore, there is evolutionary leverage for the emergence of cognitive processes dedicated to processing the social role of touch from infancy onward. Such processes need to be able (i) to react adaptively to the source of touch, (ii) specifically for tactile stimulations that carry affiliative content. The first step to achieve these two functions is to detect relevant kinds of social touch.

At the neural level, the identification of affiliative touch is likely to involve CTs, a class of unmyelinated afferents present in the hairy skin (McGlone et al., 2014; Morrison et al., 2010). In human adults, the mean firing rate of CTs in response to touch is maximal for tactile stimulations that have the thermo-mechanical properties of caresses : a temperature of 32 °C, matching the external temperature of human skin (Ackerley et al., 2014b), and an intermediate velocity between 1–10 cm/s (Ackerley et al., 2014b; Löken). CTs also respond to very low indentation forces in the range 0.3–2.5 mN (Cole et al., 2006; Vallbo et al., 1999) which also correspond to a gentle caress.

CTs project to a network of cerebral regions that play an active role in social cognition, including the posterior insula, posterior superior temporal cortex, medial prefrontal cortex, and dorsal anterior cingulate cortex (Björnsdotter et al., 2014; Gordon et al., 2013; Olausson et al., 2002, 2008; Van de Winckel et al., 2013; Voos et al., 2012). Strokes of intermediate velocity, which elicit the highest mean firing rates in CTs, are also rated as more pleasant than slower or faster strokes by children, adolescents, and adults (Ackerley et al., 2014a; Croy et al., 2017; Essick et al., 2010; Löken et al., 2009; Sehlstedt et al., 2016). Thus, CTs may act as an entry point for an early developing system dedicated to processing affiliative touch. In line with this proposal, Fairhurst et al. (2014) found that strokes of intermediate velocity (3 cm/s) elicit a larger decrease in heart rate and longer individual gazes towards the stroking stimulus than slower or faster strokes in nine-month-old human infants. Furthermore, from two months of age, strokes of intermediate velocity (3 cm/s) elicit more activity in the temporal and in the insular cortex than faster strokes (20 cm/s) (Jönsson et al., 2018). In short, cerebral, physiological and behavioral measures suggest that strokes of intermediate velocity have a special status for infants, and that they trigger activity in brain areas linked to socio-affective processing.

We built upon these previous results to test whether infants’ response to interpersonal touch is (i) modulated by the source of tactile stimulation, (ii) specifically for tactile stimulations that are known to elicit maximal mean firing rates in CTs. We measured the heart rate of three groups of nine-month-olds while their legs were stroked with a brush. The participants were stroked at a different speed in each group (0.3 cm/s, 3 cm/s, 30 cm/s). Depending on condition, the person who acted as the source of touch was either the participants’ caregiver (“parent condition”) or an unfamiliar experimenter (“stranger condition”). In fact, another experimenter blind to the Identity condition always delivered the strokes, thus ensuring that the mechanical properties of the tactile stimulation were kept constant across treatments. A cardiac deceleration during touch is usually interpreted as indicative of relaxation (Aureli et al., 1999; Drescher et al., 1985; Triscoli et al., 2017; Weiss, 1992). Here, to test whether infants are sensitive to the source of touch, we compared their heart rate deceleration in the parent and in the stranger condition. Furthermore, to assess the role of CTs in infants’ sensitivity to the source of touch, we evaluated whether the effect of Identity (parent vs. stranger) is stronger when strokes are given at an intermediate (i.e., CT-optimal) velocity rather than at a slow or fast velocity.

## 2 Material and methods

### 2.1 Participants

We tested 9-month-old participants. By this age, infants’ aversive reaction to strangers is well established (Sroufe, 1977). Furthermore, the cerebral response to affective touch of infants younger than 8 months of age might still be immature (Kida and Shinohara, 2013; Miguel et al., 2017; Pirazzoli et al., 2019). Sample sizes were modeled after those in comparable studies (Fairhurst et al., 2014). Given the age of the participants and our experiment’s duration (approximately 10 min), it was not possible to collect within-subject data across all three velocity conditions (see e.g., Sailer and Ackerley, 2019; Von Mohr et al., 2017 for other studies comparing the effect of social touch between subjects). As a result, each participant was only tested once, in a single velocity condition. Sixteen nine-month-old infants for each group were retained in the analysis (slow condition: 7 girls, 9 boys; *M_age_* = 275 days; *SD* = 18 days; age range: 242–305 days; CT-optimal condition: 9 girls, 7 boys; *M_age_* = 280 days; *SD* = 17 days; age range: 244–305 days; fast condition: 9 girls, 7 boys; *M_age_* = 275 days; *SD* = 22 day; age range: 242–305 days). Twenty-five additional participants were excluded from the analysis because the participant became too distressed to make it possible to complete data collection (9), leg movements or positions that prevented the experimenter from delivering the tactile stimulation (8), snatched electrodes (4) or technical failure (4). The research reported in this manuscript followed the guidelines of the declaration of Helsinki and was approved by an independent ethical committee for bio-medical research (CPP Sud-Est II, IRB: 00009118). The parents of all participants gave their written informed consent prior to their inclusion in the study.

### 2.2 Materials

We used two identical synthetic fiber brushes (Raphael Kaërell 8254, width: 5 cm). The first was used by the experimenter hidden behind the curtain and served to deliver the tactile stimulation. The second brush was held by an adult (either the participant’s parent or an unfamiliar experimenter) who acted as the possible visible source of touch. Heart rate responses were recorded using a 3-lead electrocardiogram (Biopac MP36, electrodes Biopac EL104). Two electrodes were placed under each clavicle, and the last one was placed on the left floating rib. The experiment was recorded by 4 different cameras (at 25 frames/s). Two ceiling cameras recorded the global scene and allowed us to confirm offline that the caregivers followed the instructions appropriately. Two additional cameras—one focused on the infant’s upper body and the other on the infant’s legs—allowed us to identify excessive movement artifacts offline. Participants were placed in an infant chair (Childwood, Seat Evolu 2, 56 × 56 × 92 cm). A large tray of plastic and pieces of opaque fabric affixed to the chair prevented participants from seeing who was touching their leg. Throughout the experiment, the experimenter that stroked the participant was hidden behind an opaque curtain located on the infant chair’s right side (sides are given from the participant’s viewpoint). A hole in the curtain enabled the hidden experimenter to brush the infants’ right shin. A video of the exact duration of one experimental block, i.e. 130 s (extracted from Baby Mozart, Baby Einstein) was played on a tablet placed approximately 40 cm away from the participant (9,7”, 24.1 × 18.5 x 0.8 cm). The same video was repeated for each block.

### 2.3 Procedure

**Fig. 1:**
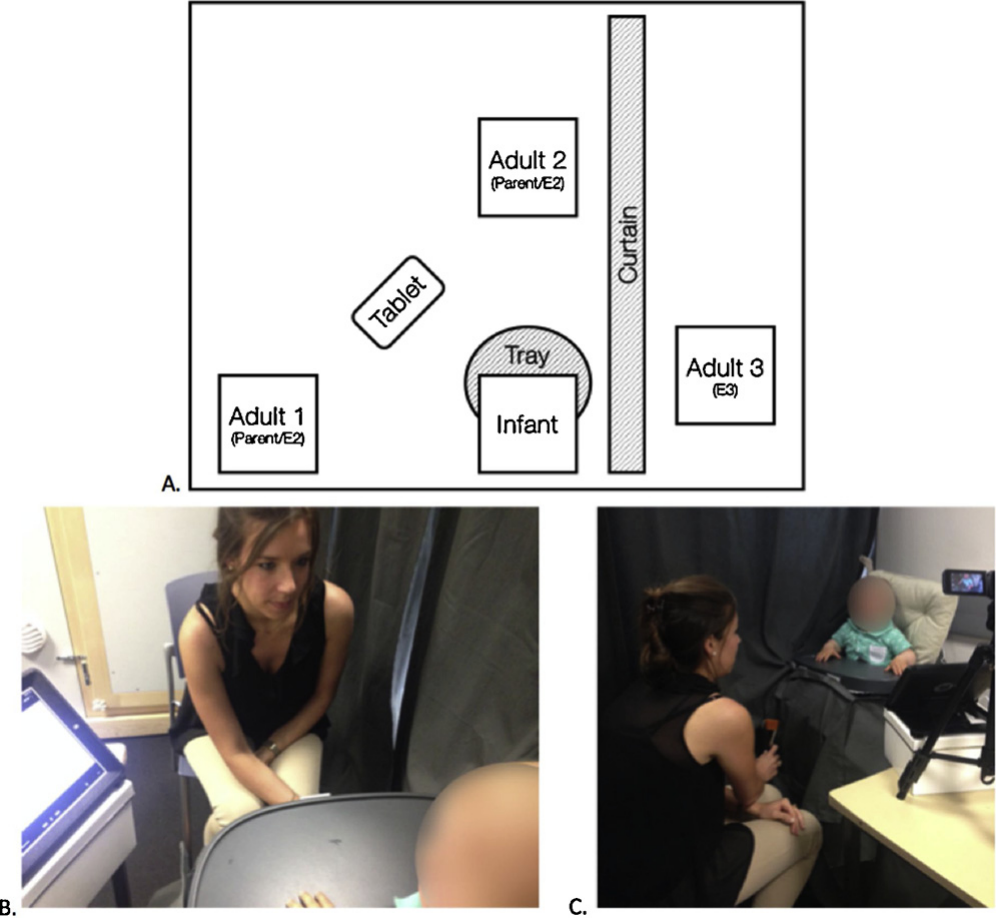
Panel A: Schematic representation of the spatial position and orientation of the participants. Each box represents a participant: either the subject (infant), the unfamiliar experimenter (E2) or the parent, and the experimenter stroking the infant’s leg (E3). A tablet displayed videos during the experiment, a plastic tray prevented infants from seeing who stroked their legs and a curtain separated the room in two parts, and allowed E3 to remain hidden from the infant’s viewpoint. Panels B and C: Pictures of the real set up. The adult is positioning the paintbrush next to the participants’ right leg, without touching the leg (brush-down position).

Prior to the experiment, a first experimenter (E1) trained caregivers to follow the experimental procedure and prepared the settings. Once the setup was ready, E1 left, and the experiment began. A second experimenter (E2, the “stranger”) entered the room. E2 did not interact with the participants prior to the experiment. The experiment began with a waiting period of 60 s, during which the parent and E2 stood on the left side of the participant. The remainder of the experiment was divided into 4 blocks. Each block began with 10 s of positioning, during which the parent and E2 moved to their respective sitting positions. One adult sat in front of the participant, and the other adult sat on the participant’s left side, each at about 60 cm from the infant chair (see Fig. 1).

The adult sitting in front of the infant placed the paintbrush next to the participants’ right leg, without touching the leg (brush-down position). When the adult was in this position, the plastic tray affixed to the infant chair prevented the participant from seeing the forearms or hands of the adult or the brush that she held. Since the infants’ leg was accessible to the adult sitting in front of the participant, it was theoretically possible for this adult to stroke the participant’s leg with the brush. Furthermore, the arms and hands of the person sitting on the left side were fully visible to the participant throughout the block, thus demonstrating that she could not stroke the infants’ leg. A piece of fabric affixed to the chair prevented the parent and E2 from seeing how the participant’s leg was stroked. The positioning was followed by three consecutive trials, each divided into four segments: resting (10 s), presentation of the brush (5 s), stimulation (10 s) and resting (20 s).

During the resting periods, the person sitting in front of the participant held the paintbrush in the brush-down position next to the participants’ leg, but no tactile stimulation was given. During the presentation of the brush (5 s), the person sitting in front of the infant showed her the paintbrush by raising it to the participant’s eye level for approximately 2 s, before placing the brush back in the brush-down position next to the participant’s legs. The presentation of the brush served to show infants that the person sitting in front of them had a brush in hand. The presentation of the brush was followed by the stimulation period (10 s), which followed the same procedure as the resting period, with one exception: during the stimulation period, a third experimenter hidden behind the curtain (E3) stroked the participant’s leg at a velocity of 0.3 cm/s (slow condition), 3 cm/s (CT-optimal condition) or 30 cm/s (fast condition). Strokes were applied on the right side of the shin region of the right leg, along an axis parallel to the tibia (following Tuulari et al., 2017). The length of the stimulated area was 3 cm. This length was chosen to allow us to run the brush over the entire stimulated area during the 10 s of stimulation for all velocities, including in the slow condition (0.3 cm/s). Given the size of CTs receptive fields in humans (mean field size 7 mm2, Wessberg et al., 2003), a length of 3 cm is sufficient to run across the receptive fields of numerous individual CTs. The stimulated area was marked out with a surgical pen prior to the experiment. Strokes were delivered back and forth, thus resulting in a single repetition of the brushing in the slow condition, 10 repetitions in the CT-optimal condition and 100 repetitions in the fast condition. The paintbrush was handheld by E3 who was trained to deliver the stimuli in all three velocities. E3 also used videos displaying the paintbrush moving at the appropriate speed as a guide, and she controlled visually that the bending of the hairs of the brush was constant across conditions (for validations of this type of procedure, see Fairhurst et al., 2014; Triscoli et al., 2013). The curtain and the additional pieces of fabric affixed to the chair prevented E3 from seeing who was sitting in front of the participant. After each stimulation, there was a 20-s resting period that served to prevent fiber fatigue.

We measured the heart rate of the infants during the stimulation phase for each trial. To have an estimate of infants’ baseline cardiac rhythm, we also measured the heart rate during the 10 s of the resting period that preceded the presentation of the brush and during the 10 s of the resting period that followed the tactile stimulation offset. For each trial, the infants’ baseline cardiac rhythm was calculated by averaging the heart rates computed over these pre- and post- stimulation resting periods. During the positioning phase at the beginning of the second, third and fourth block, the stranger and the parent exchanged roles. They swapped sitting positions, and the adult who sat in front of the infant during the preceding block gave the brush to the other adult. As a result, the stranger and the parent each sat in front of the infant for 2 blocks. Whether the stranger or the parent sat in front of the infant during the first block was counterbalanced across participants in the CT-optimal condition and in the fast condition. Due to an experimental error, in the slow condition, the caregiver sat in front of the infant during the first block for 7 participants, while the stranger sat in front of the infant during the first block for 9 participants. Two different discrete sounds indicated when adults had to show the infant the paintbrush (during the presentation of the brush phase) and when they had to swap sitting positions (during the positioning phase).

Following an anonymous reviewer’s suggestion, we also collected data on the caregiver’s attitudes towards interpersonal touch *a posteriori* (a few months after the study was run). To this end, we contacted the caregivers who participated to the experiment with the infants to ask them to fill in the Social Touch Questionnaire, which measures the respondents’ anxiety towards situations involving interpersonal touch (SQT: Wilhelm et al., 2001, twenty items, a higher score indicating a higher anxiety towards interpersonal touch), and the stroking subscale of the Parent-Infant Caregiving Touch Scale, which measures self-reports of how often the caregiver stroked her baby's back, head, tummy, arms, and legs (S-PICTS: Koukounari et al., 2015, four items, a higher score indicating a higher frequency of stroking behaviors directed towards the infant). The caregivers were asked to fill in the S-PICTS to report their behaviors when the infants were 9-month-old (i.e., when they participated to our study). Data was collected online, by sending a direct link to the questionnaires to the participants by e-mail. Out of 48 participants, 41 replied to our request and filled in the questionnaires (slow condition: *n* = 15, CT-optimal condition: *n* = 12, fast condition: *n* = 14).

### 2.4 Data analysis

After a visual inspection of the video recordings and cardiac data, we removed segments with (i) excessive movements from the participant and (ii) noisy cardiac data (the percentages of removed segments were respectively 13.37% for the slow condition, 17.36% for the CT-optimal condition, and 11.46% for the fast condition). For the remaining segments, we extracted the heart rate in heartbeats per minute (BPM) from the raw cardiac ECG data (using AcqKnowledge 4.4.2). Next, we computed the mean heart rate during stimulation and baseline for each condition (parent vs. stranger): we separately averaged heart rates during stimulation (0–10 s from stroking onset) and during baseline (10 s before the presentation of the brush and 10 s following the stimulation period). These values were then averaged across trials for each Identity condition (parent vs. stranger). The ratio of the signal change in heart rate (Hr) was calculated for each participant by computing the difference between the stimulation and baseline mean heart rates divided by the baseline mean heart rate (Loggia et al., 2011).

$$Hr=\frac{AverageHeartRate_{Stimulation}-AverageHeartRate_{Baseline}}{AverageHeartRate_{Baseline}}$$

By performing our main analyses on Hr, a baseline-corrected measure, we reduce the influence of inter-individual differences heart rate on our results. All statistical analyses reported in this paper are two-tailed. Unless specified otherwise, the data fulfilled the criteria for standard parametric analyses. Assumptions of normality were assessed with Lilliefors tests, which revealed that the data from the STQ and the S-PICTS departed from normality. Subsequently the correlations with the STQ and S-PICTS scores were analyzed using Spearman’s rho. The scores on the STQ and on the S-PICTS were not correlated (*ρ* = -0.06, *p* =  .692), thus justifying to analyze them separately. The internal consistency was good for both questionnaires (Cronbach’s α = .88 for the SQT, and α = .72 for the S-PICTS). All the statistical analyses reported in the main manuscript were performed using Statistica (version 12), with two exceptions. When data violated assumptions of homoscedasticity as assessed by Levene’s test, we used the Welch-James approximate degrees of freedom (ADF) test instead of a traditional ANOVA (Keselman et al., 2003; Lix and Keselman, 1995; Welch, 1951). These analyses were conducted in R using the package ‘welchADF’ (https://cran.r-project.org/web/packages/welchADF/index.html). Partial correlation analyses were performed in R using the ppcor package (v.1.1, Kim, 2015).

## 3 Results

A mixed-model ANOVA using the Welch-James ADF procedure on mean signal change in heart rate from baseline to test (Hr) with Identity (parent vs. stranger) as a within-subject factor and Velocity (slow, CT-optimal, fast) as a between-subject factor revealed an interaction between Identity and Velocity (*F*(2, 27.91) = 3.59, *p* =  .041). The ANOVA on mean Hr revealed no other significant effect. We conducted two additional ANOVAs in order to compare participants’ cardiac responses in the CT-optimal condition, with their responses in each of the other velocity conditions. In the comparison of the results from the CT-optimal condition to those from the slow condition, a mixed-model ANOVA using the Welch-James ADF procedure on mean Hr with Identity (parent vs. stranger) as a within-subject factor and Velocity (CT-optimal vs. slow condition) as a between-subject factor revealed a main effect of Identity (*F*(1, 23.64) = 4.735, *p* =  0.040) and an interaction between Identity and Velocity (*F*(1, 23.64) = 6.139, *p* =  0.021). Additionally, in the comparison of the results from the CT-optimal condition and those from the fast condition, a mixed-model ANOVA on mean Hr with Identity (parent vs. stranger) as a within-subject factor and Velocity (CT-optimal vs. fast condition) as a between-subject factor also revealed an interaction between Identity and Velocity (*F*(1, 30) = 5.933, *p* =  0.021, *η^2^_p_* = 0.165). Thus, the effect of Identity on mean heart rate and on mean Hr varied depending on the velocity of the tactile stimulation, and it was significantly different for strokes of intermediate, i.e., CT-optimal velocity rather than for slow or fast velocity.

**Fig. 2:**
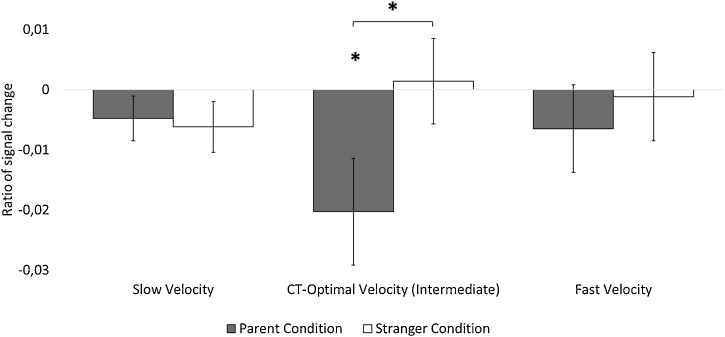
Mean ratio of signal change in heart rate from baseline to test (SEM) for all stroking velocity condition (slow, CT-optimal and fast). *: *p* <  0.05.

As Fig. 2 shows, in the CT-optimal velocity condition, Hr was significantly lower in the parent condition (*M_Hr_* = −0.020, *SD* = 0.035) than in the stranger condition (*M_Hr_* = 0.001, *SD* = 0.029, *t*(15) = 2.67, *p* = .017, *d* = 0.67, paired Student t-test). In contrast, Identity (parent vs. stranger) had no significant effect on Hr in neither the slow condition (*M_Hr_* = −0.005, *SD* = 0.015 vs. *M_Hr_* = −0.006, *SD* = 0.017, *t*(15) = −0.31, *p* = 0.76, *d* = −0.077, paired Student t-test) nor the fast condition (*M_Hr_* = −0.007, *SD* = 0.029 vs. *M_Hr_* = −0.012, *SD* = 0.029, *t*(15) = −0.69, *p* = 0.50, *d* = −0.17, paired Student t-test).

Planned post hoc analyses revealed that the mean Hr was significantly below 0, thus indicating a heart rate deceleration from baseline to stimulation only in the parent condition of the CT-optimal velocity condition (*t*(15) = −2.29, *p* = 0.037, *d* = −0.57, univariate Student t-test). In contrast, the mean value of Hr did not differ significantly from 0 in the stranger condition of the CT-optimal velocity condition (*t*(15) = 0.20, *p* = 0.85, *d* = 0.049, univariate Student t-test). Furthermore, the mean value of Hr did not differ significantly from 0 in any of the Identity conditions in the slow condition (parent condition: *t*(15) = −1.29, *p* = 0.22, *d* = −0.32; stranger condition: *t*(15) = −1.48, *p* = 0.16, *d* = −0.37, univariate Student t-tests) or in the fast condition (parent condition: *t*(15) = −0.89, *p* = 0.39, *d* = −0.22; stranger condition: *t*(15) = −1.58, *p* = 0.13, *d* = −0.40, univariate Student t-tests).

Complementary analyses also confirmed that our main results remained significant after controlling for measures of infants’ looking behaviors towards their parent and towards the stranger, and after controlling for the caregivers’ scores on the STQ, and on the S-PICTS (see Supporting Information Complementary Results and Analyses).

Fairhurst et al. (2014), reported a significant positive correlation between the caregiver’s STQ score and the infant’ cardiac response to strokes of intermediate, i.e., CT-optimal, velocity (*r* = .56, *p* =  .02), but not for strokes of slow- (*r* = .20, *p* =  .42) or fast-velocity (*r* = .14, *p* =  .58). In a complementary analysis, we sought to replicate this result conceptually by assessing the correlations between the infant’s cardiac response (mean *Hr*) and the caregiver’s score for each of the questionnaires, and for each Velocity condition (slow, CT-optimal and fast). Pre-planned analyses revealed a significant positive correlation between infants’ Hr mean response and caregivers’ STQ score in the CT-optimal condition (*ρ* = .77, *p* =  .004) but not in the slow or in the fast velocity conditions (respectively *ρ* = .35, *p* =  .201 and *ρ* = .14, *p* =  .625). These results dovetail with those reported by Fairhurst et al. (2014), and they suggest that the greater the caregiver’s anxiety towards social touch, the lower the infant’s heart rate deceleration in response to CT-optimal, intermediate velocity, touch. Furthermore, there was a marginally significant negative correlation between the infant’s mean Hr and the caregiver’s S-PICTS score in the CT-optimal condition (*ρ* = −0.55, *p* = 0.067) but not in the slow or in the fast velocity conditions (respectively *ρ* = .068, *p* = 0.811 and *ρ* = .309, *p* = 0.283). These results suggest that the more interactions and tactile stimulation between caregiver and infants there are, the greater the infant’s heart rate deceleration in response to CT-optimal, intermediate velocity, touch. Last, the partial correlation between the caregiver’s SQT scores and the infants’ mean Hr remained significant while controlling for the self-reported stroking behaviors directed towards the infant measured by the S-PICTS (*ρ* = .48, *p* = 0.020).

In addition, we ran a series of exploratory analyses in order to examine a possible connection between the caregiver’s self-reported tactile behaviors and attitude towards interpersonal touch, and the infant’s differential response to touch depending on the person acting as the source of the touch (parent vs. stranger). This exploratory analysis revealed no significant results (see Supporting Information Complementary Results and Analyses).

## 4 Discussion

In the CT-optimal condition of our experiment, infants’ response to strokes of intermediate velocity did not just depend on their mechanical properties. It varied depending on the possible source of the touch, thus dovetailing with data from adults (Gazzola et al., 2012; Scheele et al., 2014). In short, our results reveal that human infants do not treat interpersonal touch as a purely mechanical event, and that they react to its social source. This type of contextual modulation plays a crucial social function, by allowing touch to regulate social interactions and relationships between individuals. This function can be fulfilled by at least two different kinds of—mutually non-exclusive—cognitive mechanisms. First, top-down processes can influence the evaluation of touch (e.g., McCabe et al., 2008). For example, infants could evaluate more positively a tactile stimulation because they identify its source as their caregiver (for evidence for representations of caregiving relationships in infants, see Johnson et al., 2010; Johnson, Dweck, & Chen, 2007). Second, affective priming can modulate participants’ response to touch. In our case, infants could evaluate the hedonic value of intermediate velocity touch more positively when it is paired with a pleasant or familiar visual stimulus — the face of their caregiver (for comparable effects in adults, see Ellingsen et al., 2014, 2016; Croy et al., 2014; for effect of familiarity on the processing of caregivers’ faces in infants, see Kahana-Kalman and Walker-Andrews, 2001). Regardless of the exact cause of the different reactions of the infants in the caregiver and stranger condition, these differences reveal that the sensory and affective component of touch interacts with a sensitivity to the identity of the source of touch from infancy onward.

The integration of tactile information with other perceptual inputs plays a key role in the formation of a representation of one’s own body from birth onward and throughout infancy (Bremner et al., 2008; Filippetti et al., 2013, 2014; Filippetti et al., 2015; Freier et al., 2016; Rigato et al., 2014; Zmyj et al., 2011). Our results reveal that a multi-sensory interaction is also central to infants’ response to interpersonal touch. The participants’ visual, auditory and olfactory environment was identical during the baseline and during the phase of tactile stimulation across velocity conditions. Therefore, the effect of Identity that we observed specifically for strokes of intermediate velocity was likely driven by the interaction between tactile and visual information.

We found that the modulation of infants’ response to the identity of the source of touch was stronger for strokes of CT-optimal velocity than for faster or slower strokes. This result suggests that CTs, and the network of brain area upon which they project, may play a central role in infants’ sensitivity to the source of interpersonal touch. More specifically, in adults the mean firing rate of individual C-Tactile afferents is known to be higher for stimulations of intermediate, rather than slow or fast velocity, and it correlates with explicit ratings of the pleasantness of caresses (Ackerley et al., 2014a; Löken et al., 2009). Therefore, the mean firing rate of CTs (as opposed to the number of spikes elicited in CTs) is a plausible candidate neural code for the identification of pleasant touch by infants in our experiment.

In Fairhurst et al. (2014), infants’ heart rate decelerated from baseline in response to a tactile stimulation delivered at intermediate speed by an experimenter. In contrast, we did not observe a similar deceleration in our study. One possible explanation for this difference could be that in our case, the experimenter who acted as the “stranger” did not interact at all with the participants before the experiment. Moreover, in Fairhurst et al.’s study the experimenter was located behind the participants and infants actually had to turn their heads and bodies to view the experimenter holding the brush used for stroking. This may have reduced the ‘salience’ of the source of touch. In addition, infants in Fairhurst et al.’s study were sitting in a seat on their parents’ laps. While the seat prevented the parent from directly touching her infant, it may still have created a sense of caregiver presence in infants. Thus, the difference between our results and those of Fairhurst et al. (2014) may be explained by the fact that in our stranger condition the person acting as the source of touch (i) was salient and (ii) was a complete stranger.

Infants’ heart rate deceleration in reaction to CT-optimal velocity strokes tended to correlate with caregivers’ attitudes towards interpersonal touch measured by the SQT (as in Fairhurst et al., 2014), and with self-reports of caregiving stroking behaviors (measured by the S-PICTS). These correlations have to be interpreted with caution, since we merely collected self-reports from parental questionnaires. Yet, they suggest that (i) infants’ cardiac reaction to strokes of CT-optimal velocity varies and that (ii) it is stronger in infants whose caregivers have low social anxiety towards touch, and engage frequently in caregiving stroking behavior directed towards the infant. Furthermore, the correlation between infants’ heart rate deceleration in response to CT-optimal velocity strokes and caregivers SQT scores remained significant after controlling for caregivers S-PICTS scores. This additional result suggests tentatively that part of the relationships between the caregivers’ attitude towards interpersonal touch and the infants’ cardiac reaction to CT-optimal velocity strokes might be independent from the infants’ experience with parental stroking behaviors.

Touch has been argued to play a key role in building a representation of the bodily self (Bremner and Spence, 2017; Filippetti et al., 2013; Meltzoff et al., 2018; Saby et al., 2015), which in turn is crucial to distinguish oneself from others, engage in social interaction, and predict and interpret the behaviors of others (De Vignemont, 2014; Meltzoff, 2007; Müller et al., 2017). How the modulation of infants’ responses to the source of interpersonal touch that we observed in our study builds upon a representation of the interacting bodily and social selves is an important question for future research. More generally, more work on touch is needed to understand the early ontogeny of social cognition. Currently, the overwhelming majority of studies on early social cognition focus on the role of visual (and to some minor extent auditory) inputs. Yet, touch serves social and communicative functions from the first year of life and it is a privileged route for early social interactions between caregivers and infants. Moreover, interpersonal touch is central to the social life of humans and non-human primates and is processed by specific channels that are likely to contribute to social cognition (such as CTs and the brain areas upon which they project). Finally, as our data suggest, human infants do not treat interpersonal touch as a purely mechanical event, and they react to its social source.

## Author note

The authors declare no conflict of interest.
