# Supplementary material for: Infants discriminate the source of social touch at stroking speeds eliciting maximal firing rates in CT-fibers
Source: Dev Cogn Neurosci. 2019 Mar 19;36:100639. doi: 10.1016/j.dcn.2019.100639 (PMC6969234; doi:10.1016/j.dcn.2019.100639)
Supplement: Supplementary file 1 [file mmc1.docx]

**Supporting Information**

**Complementary Results and Analyses**

**Complementary Confirmatory Analyses**

In order to assess the robustness of our results, we conducted complementary analyses to ensure that our main results remained significant after controlling for measures of infants’ looking behaviors towards their parent and towards the stranger, and after controlling for the caregivers’ scores on the STQ, and on the S-PICTS. For these analyses, **l**inear mixed model regression analyses (LMM) were performed in R with the packages lme4 (version 1.1-7, Bates, Mächler, Bolker, & Walker, 2015) and lmerTest (version 3.0-1, Kuznetsova, Brockhoff, & Christensen, 2017). To explicitly take into account repeated measures, all mixed models included subject identity as a random intercept. We used likelihood ratio tests (LRTs) to evaluate model fit by assessing whether the inclusion of certain predictors significantly reduced residual variance.

**Analyses Taking Looking Behaviors Into Account**

In a complementary analysis, we assessed whether increased looking towards one of the adults during the stimulation might be a possible mechanism by which cardiac responses are influenced. We coded frame by frame the duration that infants spent looking at their parent or at the stranger, during the baseline and stimulation periods of each segment kept in the analysis of heart rates (using QuickTime Player version 7.7.9). All the data was coded by the first author and a second coder unaware of the hypotheses of the study double-coded 25% of videos in each velocity condition (Pearson’s *r* = .88, *p* < .001).

In a first basic analysis, we computed “Lt_raw_” for each participant by calculating the average duration that infants spent looking towards the adult acting as the source of the touch (i.e., the parent in the Parent condition and the experimenter in the Stranger condition) in each Identity condition (parent vs. stranger) during the stroking phase. Detailed descriptive statistics for Lt_raw_ are given in table S1. First, we analyzed the effect of Identity and Velocity on Lt_raw._  To this end, we ran a linear mixed model with Lt_raw_ as our dependent variable, with a model including subject identity as a random intercept, and fixed effects of Identity (parent vs. stranger), Velocity (slow, CT-Optimal, fast) and of the interaction between Identity and Velocity. Only Identity was a significant predictor in the resulting model (*F*(1, 45) = 16.11, *p* < .001). We speculate that participants looked longer towards the stranger as the source of the tactile stimulation because this person was less familiar. As per request from an anonymous reviewer, we also assessed whether Lt_raw_ differed significantly across identity conditions in the CT-optimal velocity group. There was no significant difference in average Lt_raw_ between the caregiver and the stranger conditions in the CT-optimal velocity stroking group (caregiver: *M* = 1.65s, *SD* = 1.57; stranger: *M* = 2.08s, *SD* = 1.43, *t*(15) = 1.09, *p* = .29, *d* = 0.27, paired Student t-test).

Next, we tested whether the effects that we observed on Hr remained significant in analyses taking into account Lt_raw_. First, we assessed the robustness of the interaction between Identity (parent vs. stranger) and Velocity (slow, CT-optimal, fast). We ran a linear mixed model with Hr as our dependent variable, and with an initial model including subject identity as a random intercept, and fixed effects of Identity (parent vs. stranger), Velocity (slow, CT-optimal, fast) and Lt_raw_.. Adding the fixed effect of the interaction between Identity and Velocity to the model improved significantly its goodness of fit by a LRT (*χ^2^*(2) = 7.30, *p* = 0.026).

Second, we assessed the robustness of the effect of Identity (parent vs. stranger) on Hr observed in the CT-optimal velocity condition. We started this analysis by running a linear mixed model with Hr as our dependent variable, and with an initial model including only subject identity as a random intercept, and a fixed effect of Lt_raw_, analyzing only the data collected in the CT-optimal velocity condition. Next, we added a fixed effect of Identity to the model, which improved significantly its goodness of fit by a LRT (*χ^2^*(1) = 6.25, *p* = 0.012).

In the second series of analyses, we controlled for infants’ change in looking behaviors from the baseline to the stimulation period. In order to implement this analysis, we computed infants’ preferential looking towards the adult acting as the source of the touch (i.e., the parent in the Parent condition and the experimenter in the Stranger condition). This measure of preferential looking time was computed by dividing the duration spent looking towards the adult acting as the source of the touch by the sum of the duration spent looking towards the parent and towards the stranger (e.g., Della Longa, Gliga & Farroni, in press). Preferential looking measures were computed separately for the baseline, and for the stimulation periods, for each trial and each participant. These measures were then averaged across trials for each Identity condition (parent vs. stranger), for each participant. Next, we computed a measure of signal change in infants’ looking preference from baseline to test (Lt_change_). This measure of signal change in looking preference was calculated by subtracting the average looking preference towards the adult acting as the source of the touch during the baseline to the average looking preference towards the adult acting as the source of the touch during the test. Lt_change_ was calculated separately for each participant, in each Identity condition (parent vs. stranger).

Detailed descriptive statistics for Lt_change_ are given in table S2. In a preliminary analysis, we assessed the effect of Identity and Velocity on Lt_change._  To this end, we ran a linear mixed model with Lt_change_ as our dependent variable, with a model including subject identity as a random intercept, and fixed effects of Identity (parent vs. stranger), Velocity (slow, CT-Optimal, fast) and of the interaction between Identity and Velocity. In the resulting model, no main effect or interaction was significant. As per request from an anonymous reviewer, we also assessed whether Lt_change_ differed significantly across identity conditions in the CT-optimal velocity group. There was no significant difference for Lt_change_ between the caregiver and the stranger conditions in the CT-optimal group (caregiver: *M* = 0.022, *SD* = 0.38; stranger: *M* = 0.044, *SD* = 0.25, *t*(15) = 0.18, *p* = .86, *d* = 0.046, paired Student t-test).

Next, we assessed whether the interaction between Identity (parent vs. stranger) and Velocity (slow, CT-optimal, fast) could be explained by changes in the infants’ preferential looking towards the adults from the baseline to the stimulation phase. We ran a linear mixed model with Hr as our dependent variable, and with an initial model including subject identity as a random intercept, and fixed effects of Identity (parent vs. stranger), Velocity (slow, CT-optimal, fast) and Lt_change_. Adding a fixed effect of the interaction between Identity and Velocity to the model improved significantly its goodness of fit by a LRT (*χ^2^*(2) = 10.49, *p* = 0.005).

Second, we assessed the robustness of the effect of Identity (parent vs. stranger) on Hr observed in the CT-optimal velocity condition. We started this analysis by running a linear mixed model with Hr as our dependent variable, with an initial model including only subject identity as a random intercept and a fixed effect of Lt_change_, and analyzing only the data collected in the CT-optimal velocity condition. Next, we added a fixed effect of Identity to the model, which improved significantly its goodness of fit by a LRT (*χ^2^*(1) = 6.33, *p* = 0.012).

In sum, our main conclusions remain supported by analyses controlling for infants’ looking behaviors.

**Analyses Taking The Caregivers’ Self-reported Tactile Behaviors And Attitudes Towards Interpersonal Touch Into Account**

We conducted further analyses to evaluate the robustness of our main results, while taking into account the caregivers’ self-reports of tactile behaviors and attitudes towards interpersonal touch. These analyses were completed on the sample of participants who replied to our request to fill in the Social Touch Questionnaire —STQ (Wilhelm, Kochar, Roth, & Gross, 2001)— and the stroking subscale of the Parent Infant Caregiving Touch Scale —S-PICTS (Koukounari, Pickles, Hill, & Sharp, 2015)— (slow condition: *n* = 15, CT-optimal condition: *n* = 12, fast condition: *n* =14). First, a preliminary analysis indicated that there was no effect of velocity condition (slow; CT-optimal; fast) neither on STQ scores (*H(2)* = 3.48, *p* = .176, Kruskal-Wallis test), nor on S-PICTS scores (*H(2)* = 3.22, *p* = .190, Kruskal-Wallis test), thus confirming that self-reports of attitudes towards interpersonal touch were comparable across the three velocity groups. Detailed descriptive statistics for STQ and S-PICTS scores are given in table S3.

Second, we assessed the robustness of the interaction between Identity (parent vs. stranger) and Velocity (slow, CT-optimal, fast). We ran a linear mixed model with Hr as our dependent variable, and with an initial model including subject identity as a random intercept, and fixed effects of Identity (parent vs. stranger), Velocity (slow, CT-optimal, fast) and of the caregivers’ scores on the STQ and on the S-PICTS. Adding the interaction between Identity and Velocity to this model improved significantly its goodness of fit by a LRT (*χ^2^*(2) = 9.84, *p* = .007).

Third, we assessed the robustness of the effect of Identity (parent vs. stranger) on Hr observed in the CT-optimal velocity condition. We started this analysis by running a linear mixed model with Hr as our dependent variable, and with subject identity as a random intercept, and fixed effects of the caregivers’ scores on the STQ and on the S-PICTS, analyzing only the data collected in the CT-optimal velocity condition. Next, we added the fixed effect of Identity to the model, which improved significantly its goodness of fit by a LRT (*χ^2^*(1) = 9.51, *p* = 0.002).

In sum, our main conclusions remain supported by analyses controlling for the caregivers’ tactile behaviors and attitudes towards social touch, measured by the STQ and the S-PICTS.

**Relations Between The STQ and The S-PICTS Scores And The Infant’s Differential Cardiac Response To Touch Depending on Identity Condition**

We ran a series of exploratory analyses in order to examine a possible connection between the caregiver’s STQ and S-PICTS scores, and the infant’s differential response to touch depending on the Identity of the person acting as the source of the touch (parent vs. stranger). To this end, we computed the difference between the mean Hr in the parent condition minus the mean Hr in the stranger condition, for each of the participants. We assessed the correlations between this Hr differential score and the caregivers’ STQ and PICTS scores. Since these analyses were exploratory, we applied a Bonferroni correction for 3 comparisons (one per Velocity condition) to our *p*-values. These analyses revealed no significant correlation between the infants’ Hr differential scores and caregivers’ SQT score in any of the velocity conditions (CT-optimal: *ρ* = .24, *p_corr_* = 1; slow: *ρ* = .53, *p_corr_*  = .13; fast : *ρ* = .18, *p_corr_*  = 1). Similarly, we observed no correlation between the infant’s Hr differential score and the caregiver’s S-PICTS score in any of the velocity conditions (CT-optimal: *ρ* = -.12, *p_corr_* = 1; slow: *ρ* = .104, *p_corr_*  = 1; fast: *ρ* = .52, *p_corr_* = .162).

| Lt_raw_/Velocity |  | Slow | CT-Optimal | Fast |
| --- | --- | --- | --- | --- |
| Caregiver | mean | 0.74 | 1.65 | 1.26 |
|  | SD | 0.69 | 1.57 | 0.88 |
| Stranger | mean | 1.73 | 2.08 | 2.62 |
|  | SD | 1.51 | 1.43 | 2.21 |

Table S1. Descriptive statistics of Lt_raw_ (in seconds) per Velocity condition (slow, CT-optimal and fast) and per Identity condition (caregiver and stranger).

| Lt_change_/Velocity |  | Slow | CT-Optimal | Fast |
| --- | --- | --- | --- | --- |
| Caregiver | mean | 0.20 | 0.022 | 0.16 |
|  | SD | 0.41 | 0.38 | 0.29 |
| Stranger | mean | -0.0076 | 0.044 | 0.054 |
|  | SD | 0.27 | 0.25 | 0.26 |

Table S2. Descriptive statistics of Lt_change_ per Velocity condition (slow, CT-optimal and fast) and per Identity condition (caregiver and stranger).

| Questionnaires/Velocity |  | Slow | CT-Optimal | Fast |
| --- | --- | --- | --- | --- |
| SQT | mean | 27.40 | 33.17 | 36.00 |
|  | SD | 11.54 | 12.35 | 11.71 |
| S-PICTS | mean | 16.93 | 17.25 | 15.71 |
|  | SD | 2.47 | 1.87 | 2.34 |

Table S3. Descriptive statistics of the two Questionnaires (SQT and S-PICTS) per Velocity condition (slow, CT-optimal and fast).

**References**

Bates, D., Mächler, M., Bolker, B. M., & Walker, S. C. (2015). Fitting linear mixed-effects models using lme4. *Journal of statistical software*, *67*(1), 1-48.

Della Longa, L., Gliga, T., & Farroni, T. (in press). Tune to touch: Affective touch enhances learning of face identity in 4-month-old infants. *Developmental Cognitive Neuroscience*. https://doi.org/10.1016/j.dcn.2017.11.002

Fairhurst, M. T., Löken, L., & Grossmann, T. (2014). Physiological and behavioral responses reveal 9-month-old infants’ sensitivity to pleasant touch. *Psychological Science*, *25*(5), 1124–1131.

Koukounari, A., Pickles, A., Hill, J., & Sharp, H. (2015). Psychometric Properties of the Parent-Infant Caregiving Touch Scale. *Frontiers in Psychology*, *6*. https://doi.org/10.3389/fpsyg.2015.01887

Kuznetsova, A., Brockhoff, P. B., & Christensen, R. H. B. (2017). lmerTest package: tests in linear mixed effects models. *Journal of Statistical Software*, *82*(13), 1-26.

Wilhelm, F. H., Kochar, A. S., Roth, W. T., & Gross, J. J. (2001). Social anxiety and response to touch: incongruence between self-evaluative and physiological reactions. *Biological Psychology*, *58*(3), 181–202. https://doi.org/10.1016/S0301-0511(01)00113-2
